# Case report: The altered rate of monocytic cell death in a patient of Muckle-Wells syndrome with atypical clinical course

**DOI:** 10.3389/fped.2023.1133097

**Published:** 2023-02-16

**Authors:** Saori Murakawa, Toru Yoneda, Takayuki Hoshina, Masataka Ishimura, Koichi Kusuhara

**Affiliations:** ^1^ Department of Pediatrics, School of Medicine, University of Occupational and Environmental Health, Japan, Kitakyushu, Japan; ^2^ Department of Pediatrics, Graduate School of Medical Sciences, Kyushu University, Fukuoka, Japan

**Keywords:** Muckle-Wells syndrome, cryopyrin-associated periodic syndrome, NLRP3 gene mutation, hearing loss, monocytic cell death

## Abstract

Muckle-Wells syndrome (MWS) is an autosomal dominant autoinflammatory disease recognized as the intermediate phenotype of cryopyrin-associated periodic syndrome (CAPS) caused by *NLRP3* gene mutation. It often takes a long time before the diagnosis is made because the clinical presentation of MWS is variable. We report a pediatric case who had had persistently elevated serum C-reactive protein (CRP) level since infancy and was diagnosed with MWS by the development of sensorineural hearing loss in school age. The patient had no periodic symptoms of MWS until the development of sensorineural hearing loss. It is important to differentiate MWS in patients with persistent serum CRP elevation, even if no periodic symptoms, including fever, arthralgia, myalgia and rash, are observed. Furthermore, in this patient, lipopolysaccharide (LPS)-induced monocytic cell death occurred, but to a lesser degree than has been reported in patients with chronic infantile neurological cutaneous, and articular syndrome (CINCA). Because CINCA and MWS are phenotypic variants on the same clinical spectrum, this suggests that a further large-scale study is desired to investigate the association between degree of monocytic cell death and disease severity in CAPS patients.

## Introduction

Muckle-Wells syndrome (MWS) is an autosomal dominant autoinflammatory disease recognized as the intermediate phenotype of cryopyrin-associated periodic syndrome (CAPS) caused by *NLRP3* gene mutation (1). Although MWS is characterized by recurrent fever, skin rash, arthralgias and sensorineural hearing loss, it often takes a long time before the diagnosis is made because the clinical presentation of MWS is variable (2). Some patients with the *NLRP3* mutation were free of MWS-related symptoms other than hearing loss (3). Persistent elevations of serum levels of C-reactive protein (CRP) and amyloid A are also characteristics of MWS and their elevations in the asymptomatic phase of the disease are useful for the differentiations of other autoinflammatory diseases and chronic urticaria (4).

We herein report a pediatric case who had had persistently elevated serum CRP level since infancy and was diagnosed with MWS by the development of sensorineural hearing loss in school age. The patients have had no periodic symptoms of MWS until the development of sensorineural hearing loss.

## Case presentation

A 10-year-old girl had been followed up for 9 years because of unexplained persistent elevation of serum CRP level since she was 1 year old. She had had fever and swelling in her left thigh and knee at 5, 6, 7, 8 and 10 months of age. The onset of the symptoms was always accompanied by leukocytosis (16.0–27.4 × 10^9^/L) and marked serum CRP elevation (28–150 mg/L). Although gonarthritis was suspected, no causative bacteria were detected. Symptoms that had appeared for fifth time spontaneously resolved without antimicrobial agents. The magnetic resonance imaging (MRI) showed a lobulated mass in left vastus lateralis. The lesion was completely removed by surgical intervention and was diagnosed as intramuscular venous malformation. We speculated that leukocytosis and serum CRP elevation might be caused by recurrent bleeding that led to local and systemic inflammation secondary to the extravascular accumulation of heme and heme-derived iron (5).

**Figure 1:**
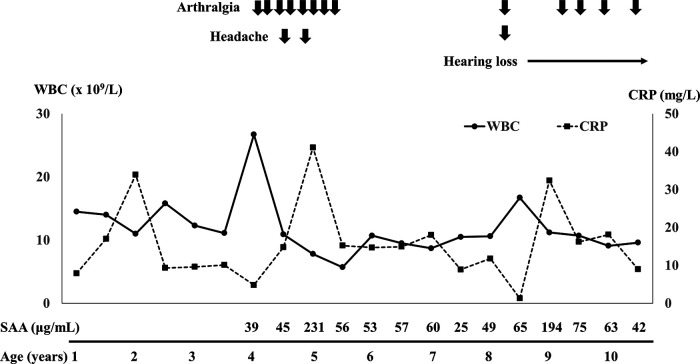
Clinical course and serial changes of laboratory data after 1 year of age. WBC, white blood cell; CRP, C-reactive protein; SAA, serum amyloid A.

**Figure 2:**
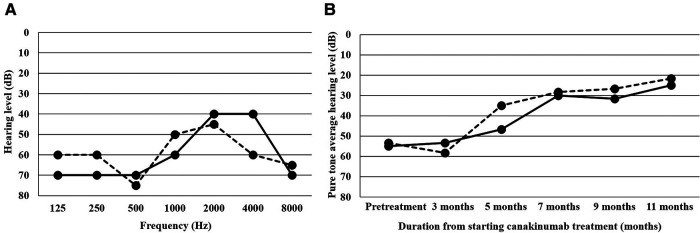
Hearing threshold in the patient with Muckle-Wells syndrome. (**A**) Hearing threshold before canakinumab therapy, (**B**) serial changes of the pure tone average for the frequencies 500, 1,000 and 2,000 Hz (three-frequency pure tone average). The solid and dashed lines indicate the hearing thresholds of right and left ears, respectively.

After the resection of the mass, no clinical symptoms, including fever and swelling in her left thigh and knee, relapsed whereas leukocytosis (around 15.0 × 10^9^/L) and mild serum CRP elevation (around 10.0 mg/L) had persisted (Figure 1). Her growth was normal. After 4 years of age, the patient irregularly complained of leg pain and headache (Figure 1). These symptoms spontaneously resolved within a few days and no abnormal findings were revealed on multiple leg MRIs between ages 4 and 8 years. Furthermore, abnormal findings were also undetected on the brain MRI taken at her 8 years old. In addition to leukocytosis and serum CRP elevation, serum amyloid A level has elevated (25–231 mg/ml, normal range <8 mg/ml) (Figure 1). Erythrocyte sedimentation rate has been normal. At 8 years of age, she became aware of hearing loss, which gradually worsened. She was diagnosed with having bilateral sensorineural hearing loss (Figure 2A). The patient was suspected to have autoinflammatory disease, based on her clinical history. A previously recognized heterozygous missense mutation in the *NLRP3* gene (c.937G > A, p.Glu313Lys, formerly known as E311K mutation) was detected in the patient by the analysis of autoinflammatory diseases-related genes, including *NLRP3*, *NLRC4*, *PLCG2* and *NLRP12* genes, using the target panel sequence (conducted in Kazusa DNA Research Institute, https://www.kazusa.or.jp). The same mutation was not detected in her mother or older sister. Her father had suffered from hearing loss but did not consent to the sample collection. She had a mild cerebrospinal fluid pleocytosis (16 cells/ml, with 37.5% of polymorphonuclear cells) and bilateral papilledema. The patient was diagnosed with Muckle-Wells syndrome. Canakinumab (2 mg/kg/dose every 8 weeks) was started after the diagnosis and, over the course of 4 months, peripheral white blood cell count and serum CRP returned to levels within normal range, from 10 × 10^5^/L to 10 × 10^4^/L and from 10.0 mg/L to 1.0 mg/L, respectively. She no longer complained of leg pain or headache and her hearing threshold gradually improved over 11 months of treatment (Figure 2B).

**Figure 3:**
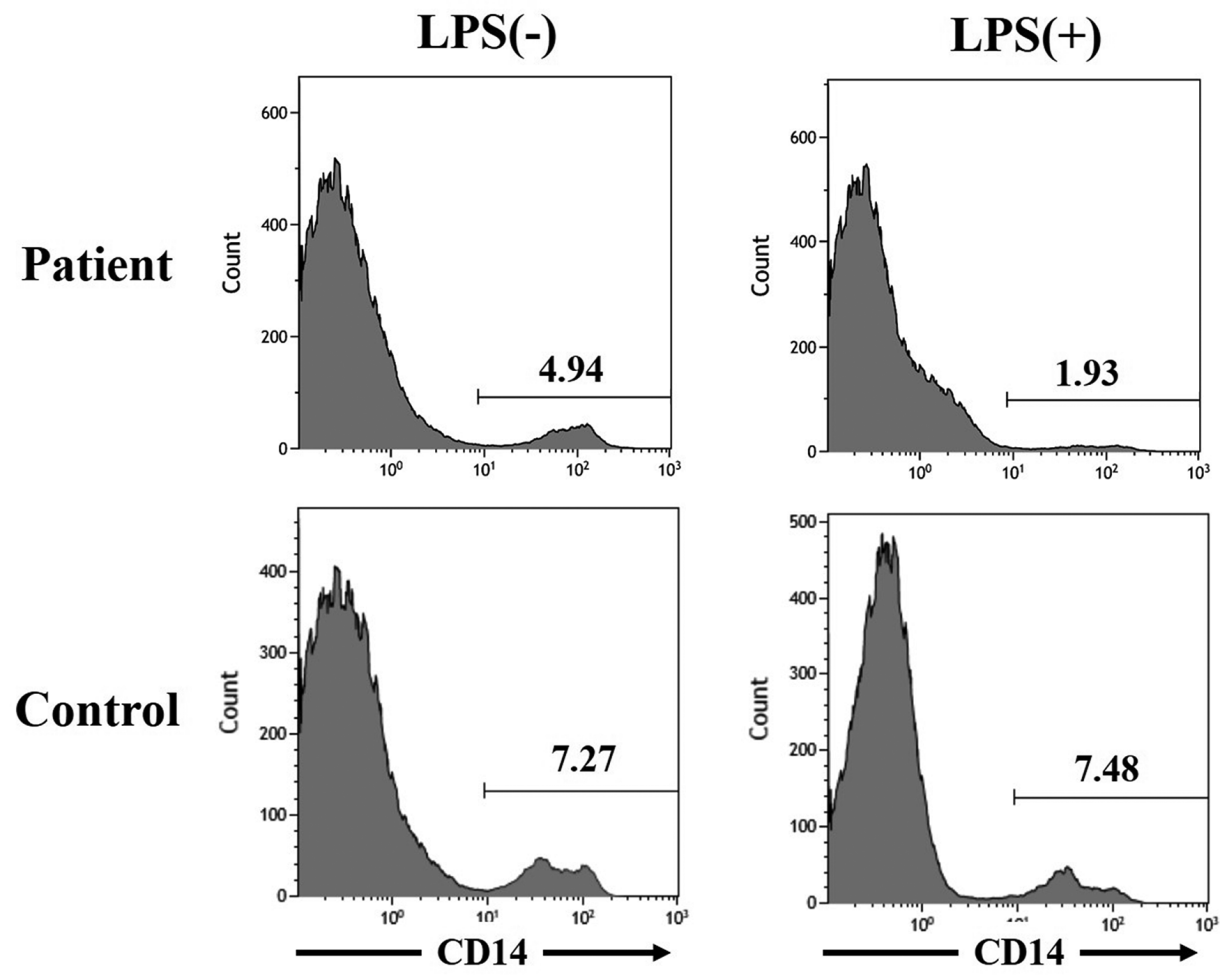
Monocytic cell death induced by lipopolysaccharide stimulation in the patient with muckle-wells syndrome. The figures of the upper left and right sides indicate the proportion of CD14^+^ cells, mainly monocytes, in peripheral blood mononuclear cells from the patient with Muckle-Wells syndrome without and with lipopolysaccharide stimulation, respectively. The figures of the lower left and right sides indicate the proportion of CD14^+^ cells, mainly monocytes, in peripheral blood mononuclear cells from an age- and sex- matched healthy child without and with lipopolysaccharide stimulation, respectively.

CAPS-associated mutations in the *NLRP3* gene induced rapid cell death of human monocytic cells (6). The previous study indicated a significant LPS-induced death of monocytes in CINCA patients (7). As the clinical course of our patient was atypical for that of MWS, we investigated the survival of monocytes with LPS stimulation in the patient as already published by our group (7). Peripheral blood mononuclear cells were incubated 24 h on the presence or absence of LPS (0.1 mg/ml) (Sigma-Aldrich, St Louis, MO, USA). The cells were stained with PE-cyanin 5.1-conjugated anti-CD14 monoclonal antibody (Beckman Coulter, Miami, FL, USA) and the proportion of monocytes was analyzed by flow cytometer (EC800 cell analyzer, Sony Corp., Tokyo, Japan). Monocytes (CD14^+^ cells) was decreased by 60.9% with LPS stimulation, which indicates a moderate cell death response compared to control (no decrease) (Figure 3). However, the reduction was less than those reported in 2 CINCA patients (99% and 98%) (7).

## Discussion

We reported a patient with MWS who had had persistent elevation of serum CRP levels and had no periodic symptoms of MWS until the development of sensorineural hearing loss. Retrospectively, the occasional leg pain and headache were considered to be MWS-related symptoms. The age at MWS diagnosis is often beyond 20 years because the clinical presentation is variable, whereas the elevation of serum CRP level is persistently observed in almost all MWS patients (2). It is important to differentiate MWS in patients with persistently elevated serum CRP levels, even in absence of periodic symptoms, such as fever, arthralgia, myalgia and rash.

The previous studies reported that patients with the *NLRP3* p.Glu313Lys variant presented more often with hearing loss, myalgia and proteinuria, whereas they had lower frequencies of febrile episodes and cold-induced urticaria and lower level of serum CRP, compared to MWS patients with other *NLRP3* gene variants (2, 8) (Table 1). Similar to other patients with the *NLRP3* p.Glu313Lys variant (2), median levels of serum CRP (14.8 mg/L) and amyloid A (56 mg/ml) in our patient were also not significantly elevated. These results suggest that MWS patients with the p.Glu313Lys variant may be more difficult to recognize and the diagnosis may be complex because of the chronicity, rather than periodicity, of their symptoms. In fact, age at diagnosis in the patients with the p.Glu313Lys mutation was older than that in those with the other mutations (2). Hearing loss is occasionally the only symptom in patients with the p.Glu313Lys mutation (3). Anti-interluekin-1 therapy is effective in stabilizing or improving hearing loss, particularly in younger patients (4). To prevent irreversible organ damage and prompt the rapid initiation of anti-interleukin-1 therapy, patients with hearing loss and the persistent elevation of serum CRP levels, like our patient, are needed to be evaluated for MWS.

**Table 1:** Clinical features in our patient and previously reported patients with Muckle-Wells syndrome with the *NLRP3* p.Glu313Lys variant.

Clinical features	Our patient	Previously reported patients (*n* = 13)^a^
Fever	No	31%
Rash	No	54%
Headache	Yes	54%
Myalgia	No	54%
Arthralgia	Yes	85%
Arthritis	No	69%
Hearing loss	Yes	92%
Eye disease	Yes	85%
Renal disease	No	77%
Age at diagnosis, years	10.8	36.6^b^

^a^ All patients were presented in the reference No. [2].

^b^ It indicates the median age.

Cryopyrin-associated periodic syndromes (CAPS), including MWS and CINCA, is characterized by overproduction of interleukin (IL)-1β, resulting from dysregulated inflammasome activity (9). Monocytes are the predominant IL-1b-producing cell population in CINCA patients, which undergo a rapid death in response to LPS stimulation (8, 9). In our patient with atypical symptoms of MWS, monocytes also decreased with LPS stimulation, but not as much as in CINCA patients (7). The results from our functional analysis were in contrast with the findings from Saito et al. (10). In their cohort, selective induction of monocytic cell death was independent of the disease severity. All enrolled patients (*n* = 12) in this study, excluding one MWS patient, had been diagnosed as CINCA, which was the most severe phenotype of CAPS (10). The mechanism of the cell death in CAPS is not clearly understood, and only one MWS patient was analyzed in the study. Although it was an analysis of only one patient, this report suggested that the LPS-induced monocytic death in the CAPS patient might be useful for evaluating disease severity and predicting the therapeutic response. A further large-scale study is desired to analyze the association between the degree of monocytic cell death and the disease severity in CAPS patients.

## Data Availability

The datasets generated for this study are available on request to the corresponding author.
